# Supplementary material for: The Effect of a Care Bundle on the Rate of Blood Culture Contamination in a General Intensive Care Unit
Source: Antibiotics (Basel). 2024 Nov 13;13(11):1082. doi: 10.3390/antibiotics13111082 (PMC11591278; doi:10.3390/antibiotics13111082)
Supplement: Supplementary file 1 [file antibiotics-13-01082-s001.zip › antibiotics-3292300-supplementary.pdf]

The effect of a care bundle on the rate of blood culture contamination in a general ICU.

SUPPLEMENTARY TABLES

Table S1. Microorganisms isolated from blood cultures during the two phases of the study. For polymicrobial bloodstream infections the different isolates have been included separately.

Pathogen	PRE phase<br>Total	POST phase<br>Total	Total<br>Total
Coagulase-negative staphylococcis	98 (32.1%)	26 (12.6%)	124 (24.3%)
K. pneumoniae	58 (19.0%)	52 (25.2%)	110 (21.5%)
A. baumanii	39 (12.8%)	41 (19.9%)	80 (15.7%)
P. aeruginosa	24 (7.9%)	17 (8.3%)	41 (8.0%)
P. stuartii	23 (7.5%)	7 (3.4%)	30 (5.9%)
Candida non-albicans	14 (4.6%)	8 (3.9%)	22 (4.3%)
E. faecalis	9 (3.0%)	12 (5.8%)	21 (4.1%)
E. faecium	7 (2.3%)	9 (4.4%)	16 (3.1%)
P. mirabilis	7 (2.3%)	3 (1.5%)	10 (2.0%)
S. maltophilia	7 (2.3%)	2 (1.0%)	9 (1.8%)
S. marcescens	7 (2.3%)	2 (1.0%)	9 (1.8%)
C. albicans	5 (1.6%)	11 (5.3%)	16 (3.1%)
P. acnes	4 (1.3%)	1 (0.5%)	5 (1.0%)
E. colis	1 (0.3%)	1 (0.5%)	2 (0.4%)
M. morganii	1 (0.3%)	0 (0.0%)	1 (0.2%)
S. aureus	1 (0.3%)	7 (3.4%)	8 (1.6%)

Table S2. Indications for blood culture in the two phases of the study.

Indication for blood culture		PRE phase	POST phase		Total
		N (%)	N (%)	P value	N (%)
Fever	Yes	716 (59.2%)	586 (58.8%)	p>0.05	1302 (59.0%)
	No	494 (40.8%)	410 (41.2%)		904 (41.0%)
WBC abnormalities	Yes	433 (35.8%)	341 (34.2%)	p>0.05	774 (35.1%)
	No	777 (64.2%)	655 (65.8%)		1432 (64.9%)
Increased CRP (>5 mg/dl)	Yes	381 (31.5%)	178 (17.9%)	p<0.001	559 (25.3%)
	No	829 (68.5%)	818 (82.1%)		1647 (74.7%)
CVC change	Yes	930 (76.9%)	977 (98.1%)	p<0.001	1907 (86.4%)
	No	280 (23.1%)	19 (1.9%)		299 (13.6%)
Previous positive BC	Yes	879 (72.7%)	789 (79.2%)	p<0.001	1668 (75.6%)
	No	330 (27.3%)	207 (20.8%)		537 (24.4%)
No BC indications	Yes	45 (3.7%)	35 (3.5%)	p>0.05	80 (3.6%)
	No	1165 (96.3%)	961 (96.5%)		2126 (96.4%)

Table S3. The distribution of blood volume in each phase

	PRE phase		POST phase		
**Total vials**	(n=2050)	(%)	(n=2187)	(%)	p
**1-4 ml**	379	18.5	0	0	<0.001+
**5-7 ml**	962	47	142	6.5	<0.001+
**8-10 ml**	665	32.4	2045	93.5	<0.001+
**>10 ml**	44	2.1	0	0	<0.001+

Table S4. Compliance to care bundle from BCs received by venipuncture

		PRE Phase (n=100, 51.3%)		POST Phase (n=95, 48.7%)		
		n	%	n	%	**P value**
**Disinfect cap of BC vials with 70% alcohol**	No	71	71.0	0	0.0	<0.001
	Yes	29	29.0	95	100.0	
**Hand hygiene before procedure**	No	62	62.0	0	0.0	<0.001
	Yes	38	38.0	95	100.0	
**Use of CHG 2% for the skin, leave time to dry**	No	60	60.0	1	1.1	<0.001
	Yes	40	40.0	94	98.9	
**Use aseptic technique for obtain, no touch critical sites**	No	57	57.0	2	2.1	<0.001
	Yes	43	43.0	93	97.9	
**Inoculate BCs first, follow other exams**	No	30	30.0	0	0.0	<0.001
	Yes	70	70.0	95	100.0	
**Overall compliance**	No	98	98.0	2	2.1	<0.001
	Yes	2	2.0	93	97.9	

Table S5. Compliance to care bundle from BCs received from CVC

		PRE Phase (n=45, 56.3%)		POST Phase (n=35, 43.8%)		
		n	%	n	%	**P value**
**Disinfect cap of BC vials with 70% alcohol**	No	25	55.6	2	5.7	<0.001
	Yes	20	44.4	33	94.3	
**Hand hygiene before procedure**	No	27	60.0	0	0.0	<0.001
	Yes	18	40.0	35	100.0	
**Use gloves (sterile or not, it depends)**	No	15	33.3	2	5.7	0.003
	Yes	30	66.7	33	94.3	
**Scrub the hub with CHG 2% for 30 sec**	No	25	55.6	0	0.0	<0.001
	Yes	20	44.4	35	100.0	
**Inoculate BCs first, follow other exams**	No	5	11.1	0	0.0	0.04
	Yes	40	88.9	35	100.0	
**Overall compliance**	No	42	93.3	4	11.4	<0.001
	Yes	5	6.7	31	88.6	

Table S6: Association between indications for BC and contaminated BC sets

Indication for blood culture		Contaminated	Negative		Total
		n (%)	n (%)	p	n
Fever	No	19 (1.8%)	1040 (98.2%)	ns	1059
	Yes	17 (2.4%)	677 (97.6%)		694
WBC abnormalities	No	16 (2.5%)	614 (97.5%)	ns	630
	Yes	20 (1.8%)	1103 (98.2%)		1123
Tachycardia or tachypnoea	No	30 (2.0%)	1459 (98.0%)	ns	1489
	Yes	6 (2.3%)	258 (97.7%)		264
CRP > 5 mg/dl	No	14 (3.2%)	428 (96.8%)	ns	442
	Yes	22 (1.7%)	1289 (98.3%)		1311
Suspected endocarditis	No	34 (2.0%)	1681 (98.0%)	ns	1715
	Yes	2 (5.3%)	36 (94.7%)		38
CVC change	No	29 (1.9%)	1502 (98.1%)	ns	1531
	Yes	7 (3.2%)	215 (96.8%)		222
Previous positive BC	No	24 (1.7%)	1348 (98.3%)	ns	1372
	Yes	12 (3.2%)	368 (96.8%)		380
No BC indications	No	2 (2.8%)	70 (97.2%)	ns	72
	Yes	34 (2.0%)	1647 (98.0%)		1681

Table S7: Association between indications for BC and contaminated or indeterminate BC sets

Indication for blood culture		Contaminated or indeterminate	Negative		Total
		n (%)	n (%)	p	n
Fever	No	67 (6.1%)	1040 (93.9%)	ns	1107
	Yes	47 (6.5%)	677 (93.5%)		724
WBC abnormalities	No	48 (7.3%)	614 (92.7%)	ns	662
	Yes	66 (5.6%)	1103 (94.4%)		1169
Tachycardia or tachypnoea	No	90 (5.8%)	1459 (94.2%)	ns	1549
	Yes	24 (8.5%)	258 (91.5%)		282
CRP > 5 mg/dl	No	40 (8.5%)	428 (91.5%)	0.016	468
	Yes	74 (5.4%)	1289 (94.6%)		1363
Suspected endocarditis	No	110 (6.1%)	1681 (93.9%)	ns	1791
	Yes	4 (10.0%)	36 (90.0%)		40
CVC change	No	93 (5.8%)	1502 (94.2%)	ns	1595
	Yes	21 (8.9%)	215 (91.1%)		236
Previous positive BC	No	80 (5.6%)	1348 (94.4%)	0.036	1428
	Yes	34 (8.5%)	368 (91.5%)		402
No BC indications	No	3 (4.1%)	70 (95.9%)	ns	73
	Yes	111 (6.3%)	1647 (93.7%)		1758

Table S8: Association between continuous variables and contaminated or indeterminate BC sets

Variable	Contaminated BC sets*	Negative BS sets*	p value	Total*
Hosp adm to BC days	18.00 (8.00-41.50)	15.00 (6.00-33.00)	0.371	15.00 (6.00-33.00)
ICU adm to BC days	10.00 (2.00-27.00)	8.00 (2.00-23.00)	0.480	8.00 (2.00-23.00)
Volume	6.00 (5.00-8.00)	8.00 (6.00-10.00)	0.007	8.00 (6.00-10.00)
Age	61.50 (45.00-71.00)	61.00 (44.00-71.00)	0.814	61.00 (44.00-71.00)
Charlson comorbidity index	2.50 (1.00-6.00)	3.00 (0 .00-5.00)	0.357	3.00 (0.00-5.00)
APACHE	16.00 (12.00-20.00)	16.00 (11.00-20.00)	0.997	16.00 (11.00-20.00)
SOFA on admission	8.00 (7.00-9.00)	9.00 (7.00-11.00)	0.296	9.00 (7.00-11.00)
SOFA on BC day	5.00 (0.00-10.00)	3.00 (0.00-9.00)	0.452	3.00 (0 .00-9.00)
Albumin on admission	3.30 (2.70-3.80)	2.90 (2.60-3.50)	0.031	3.00 (2.60-3.50)
CRP (mg/dl) on admission	5.30 (1.50-28.0)	8.40 (1.90-21.00)	0.141	8.40 (1.90-21.00)
CRP (mg/dl) on BC day	1.04 (0.00-12.70)	0.87 (0.00-12.40)	0.577	0.87 (0.00-12.40)

*Median, 25th-75th percentile

Table S9. Patient-related risk factors for contaminated BC.

Variable	Category	Contaminated	Negative	Chi-square p value	Odds ratio 995% CI)
		n (%)	n (%)		
Age (mean, SD)		65.5 (58.0-69.0)	64.0 (47.0-74.0)	0.93	n/a
Sex	Female	1 (0.55)	182 (99.45)	0.23	4.15 (0.48-35.88)
	Male	5 (2.23)	219 (97.77)		
ICU Section	Medical	5 (2.06)	238 (97.94)	0.40	0.29 (0.03-2.52)
	Surgical	1 (0.61)	163 (99.39)		
Charlson comorbidity index	n/a	4.0 (2.0-6.0)	3.0 (1.0-5.0)	0.58	n/a
Chronic dialysis	No	5 (1.27)	388 (98.73)	0.19	5.67 (0.65-54.79)
	Yes	1 (7.14)	13 (92.86)		
Asplenia	No	6 (1.49)	398 (98.51)	1.0	n/a
	Yes	0 (0.00)	3 (100.00)		
HIV (w/o AIDS)	No	6 (1.49)	396 (98.51)	1.0	n/a
	Yes	0 (0.00)	5 (100.00)		
Bone marrow transplant	No	6 (1.51)	392 (98.49)	1.0	n/a
	Yes	0 (0.00)	9 (100.00)		
Solid organ transplant	No	6 (1.50)	395 (98.50)	1.0	n/a
	Yes	0 (0.00)	6 (100.00)		
BMI<18.5	No	6 (1.49)	397 (98.51)	1.0	n/a
	Yes	0 (0.00)	4 (100.00)		
Prosthetic heart valve	No	6 (1.51)	392 (98.49)	1.0	n/a
	Yes	0 (0.00)	9 (100.00)		
Pacemaker	No	6 (1.51)	392 (98.49)	1.0	n/a
	Yes	0 (0.00)	9 (100.00)		
Vascular implant or stent	No	5 (1.30)	380 (98.70)	0.28	3.61 (0.40-32.38)
	Yes	1 (4.55)	21 (95.45)		
Orthopaedic endoprosthesis	No	6 (1.50)	393 (98.50)	1.0	n/a
	Yes	0 (0.00)	8 (100.00)		
APACHE†	n/a	19.5 (9.0-25.0)	15.0 (11.0-20.0)	0.57	n/a
SOFA on admission†	n/a	10.0 (7.0-13.0)	8.0 (7.0-11.0)	0.40	n/a
SOFA on BC day†	n/a	0.0 (0.0-5.0)	0.0 (0.0-4.0)	0.74	n/a
Albumin on admission	n/a	2.8 (2.6-3.3)0	3.0 (2.5-3.6)	0.80	n/a
CRP on admission	n/a	11.5 (3.4-34.4)	8.4 (2.0-19.1)	0.59	n/a
Albumin on BC day	n/a	0.0 (0.0-2.2)	0.0 (0.0-2.0)	0.80	n/a
CRP on BSI	n/a	0.0 (0.0-6.3)	0.0 (0.0-4.7)	1.0	n/a
Chemotherapy	No	5 (1.32)	373 (98.68)	0.36	2.67 (0.30-23.59)
	Yes	1 (3.45)	28 (96.55)		
Corticosteroids	No	5 (1.35)	365 (98.65)	0.438	2.03 (0.23-17.83)
	Yes	1 (2.70)	36 (97.30)		
Short-term CVC	No	0 (0.00)	28 (100.00)	1.0	n/a
	Yes	6 (1.58)	373 (98.42)		
Implantable CVC	No	6 (1.49)	398 (98.51)	1.0	n/a
	Yes	0 (0.00)	3 (100.00)		
Billiary or urinary drainage or stent	No	6 (1.52)	390 (98.48)	1.0	n/a
	Yes	0 (0.00)	11 (100.00)		
Peritoneal dialysis catheter	No	6 (1.48)	399 (98.52)	1.0	n/a
	Yes	0 (0.00)	2 (100.00)		
Urinary bladder catheter	No	0 (0.00)	7 (100.00)	1.0	n/a
	Yes	6 (1.50)	394 (98.50)		
Intubation	No	1 (1.75)	56 (98.25)	0.59	0.81 (0.09-7.07)
	Yes	5 (1.43)	345 (98.57)		
CRRT	No	6 (1.95)	302 (98.05)	1.0	n/a

	Yes	0 (0.00)	99 (100.00)		
Wound drainage tubes	No	5 (1.91)	257 (98.09)	0.42	0.36 (0.04-3.08)
	Yes	1 (0.69)	144 (99.31)		
Respiratory infection	No	4 (1.52)	260 (98.48)	1.0	0.92 (0.16-5.09)
	Yes	2 (1.40)	141 (98.60)		
Urinary tract infection	No	6 (1.49)	396 (98.51)	1.0	n/a
	Yes	0 (0.00)	5 (100.00)		
Intra-abdominal infection	No	6 (1.56)	379 (98.44)	1.0	n/a
	Yes	0 (0.00)	22 (100.00)		
Skin and soft tissue infection	No	6 (1.56)	379 (98.44)	1.0	n/a
	Yes	0 (0.00)	22 (100.00)		
CNS infection	No	6 (1.51)	392 (98.49)	1.0	n/a
	Yes	0 (0.00)	9 (100.00)		
CR-BSI	No	6 (1.48)	400 (98.52)	1.0	n/a
	Yes	0 (0.00)	1 (100.00)		
Surgical site infection	No	6 (1.48)	399 (98.52)	1.0	n/a
	Yes	0 (0.00)	2 (100.00)		
